# Anlotinib reverses osimertinib resistance by inhibiting epithelial-to-mesenchymal transition and angiogenesis in non-small cell lung cancer

**DOI:** 10.7555/JBR.38.20240045

**Published:** 2024-09-27

**Authors:** Liting Lyu, Xin Hua, Jiaxin Liu, Sutong Zhan, Qianqian Zhang, Xiao Liang, Jian Feng, Yong Song

**Affiliations:** 1 Department of Respiratory and Critical Care Medicine, Affiliated Jinling Hospital, Nanjing Medical University, Nanjing, Jiangsu 210002, China; 2 Department of Oncology, Affiliated Hospital of Nantong University, Nantong, Jiangsu 226001, China; 3 Southeast University Medical College, Nanjing, Jiangsu 210003, China; 4 Department of Respiratory and Critical Care Medicine, Nanjing Drum Tower Hospital, Affiliated Hospital of Medical School, Nanjing University, Nanjing, Jiangsu 210008, China; 5 Department of Respiratory and Critical Care Medicine, Nanjing Jinling Hospital, Affiliated Hospital of Medical School, Nanjing University, Nanjing, Jiangsu 210002, China; 6 Department of Oncology, Affiliated Jiangyin Hospital of Nantong University, Jiangyin, Jiangsu 214400, China; 7 Department of Pulmonary and Critical Care Medicine, Nantong Key Laboratory of Respiratory Medicine, Affiliated Hospital of Nantong University, Nantong, Jiangsu 226001, China

**Keywords:** non-small cell lung cancer, osimertinib, anlotinib, resistance, epithelial-to-mesenchymal transition

## Abstract

In the present study, we aimed to investigate whether anlotinib reverses osimertinib resistance by inhibiting the formation of epithelial-mesenchymal transition (EMT) and angiogenesis. In a clinical case, anlotinib reversed osimertinib resistance in non-small cell lung cancer (NSCLC). Therefore, we performed immunohistochemical analyses on tumor tissues from three NSCLC patients with osimertinib resistance to analyze alterations in the expression levels of EMT markers and vascular endothelial growth factor A (VEGFA) before and after the development of osimertinib resistance. The results revealed the downregulation of E-cadherin, coupled with the upregulation of vimentin and VEGFA in tumor tissues of patients exhibiting osimertinib resistance, compared with those in tissues from patients before receiving osimertinib. Subsequently, we established osimertinib-resistant (Osi-R) cell lines and found that the Osi-R cells acquired EMT features. Next, we analyzed the synergistic effects of the combination therapy to verify whether anlotinib could reverse osimertinib resistance by inhibiting EMT. The expression levels of VEGFA and tube formation were analyzed in the combination group *in vitro*. Finally, we determined the reversal of osimertinib resistance by the combination of osimertinib and anlotinib *in vivo* using 20 nude mice. The combined treatment of osimertinib and anlotinib effectively prevented the metastasis of Osi-R cells, inhibited tumor growth, exerted antitumor activity, and ultimately reversed osimertinib resistance in mice. The co-administration of osimertinib and anlotinib demonstrated synergistic efficacy in inhibiting EMT and angiogenesis in three NSCLC patients, ultimately reversing osimertinib resistance.

## Introduction

Non-small cell lung cancer (NSCLC), which exhibits a high incidence rate, poses a serious threat to human health^[1]^. Currently, treatment strategies for advanced NSCLC have entered a phase dominated by targeted therapy^[2]^ and immunotherapy^[3]^. The predominant gene mutations in NSCLC include *EGFR* mutations, *ALK* fusions, and *KRAS* mutations, while less prevalent mutations include *MET* amplification, *ROS1* fusions, *NTRK* translocations, and *RET* mutations^[4]^. The mutation rate of *EGFR* is approximately 50% in lung adenocarcinoma patients^[5]^. However, the majority of NSCLC patients inevitably develop drug resistance to osimertinib over time, with the reported possible mechanisms predominantly involving EGFR-dependent (*e.g.*, *C797X* mutation)^[6]^ and EGFR-independent pathways, including bypass activation, downstream signaling pathway activation, epithelial-mesenchymal transition (EMT), and small-cell transformation^[5,7]^. The drug-resistant patients are generally advised to undergo a second biopsy for additional mutation detection, and the corresponding combination therapies are used for those experiencing extensive progression of drug resistance^[8]^. Therefore, understanding individual resistance mechanisms and formulating rational treatment approaches may provide novel therapeutic options for such patients.

EMT, recognized as a driving force behind tumor progression, leads to both innate and acquired resistance to EGFR tyrosine kinase inhibitor (TKI) treatment^[9]^. Upon undergoing EMT, tumor cells lose the polar characteristics of epithelial cells and fail to adhere to neighboring cells^[10]^. Furthermore, the migratory and metastatic properties of tumor cells are enhanced, resulting in the acquisition of more invasive characteristics^[7]^. In NSCLC patients, the occurrence of EMT-related resistance to EGFR-TKIs is estimated to be approximately 2%^[5]^. Thus, the identification of EMT occurrence and the subsequent determination of treatment strategies may have clinical benefits for such patients. Recent advancements have indicated that a combination therapy involving antiangiogenic agents and TKIs prolongs the median progression-free survival (mPFS) and median overall survival (mOS) of patients exhibiting resistance to EGFR-TKIs^[10]^. Anlotinib, a multi-target TKI employed as a last-line treatment for advanced or metastatic NSCLC patients, exerts antiangiogenic and tumor growth-inhibitory effects by targeting the vascular endothelial growth factor receptor (VEGFR), the platelet-derived growth factor receptor (PDGFR), the fibroblast growth factor receptor (FGFR), the stem cell factor receptor (c-Kit), and other kinases^[11–12]^. The ALTER 0303 trial compared anlotinib with placebo in advanced NSCLC patients who received at least two lines of treatment, showing that anlotinib effectively extended the mOS by 3.30 months (9.60 months *vs.* 6.30 months, *P* < 0.05), compared with the placebo^[13]^, highlighting promising clinical applications for anlotinib in treating NSCLC.

Recently, clinical observations have indicated that the addition of anlotinib provides clinical benefits to patients who have become resistant to osimertinib^[14]^. However, few studies have elucidated the specific mechanism of the reversal of osimertinib resistance by anlotinib. The present study aimed to investigate whether anlotinib could reverse the EMT-induced osimertinib resistance, which might offer a novel clinical diagnostic and therapeutic strategy for the management of osimertinib resistance in NSCLC patients.

## Materials and methods

### Cell lines

PC9 and HCC827 cell lines were purchased from the American Type Culture Collection (ATCC, Manassas, VA, USA). The *EGFR* exon 19 deletion (*EGFR*-19del) cell lines, including PC9 and HCC827, were cultured in RPMI 1640 medium supplemented with 10% fetal bovine serum (FBS; Cat. #FSD500, ExCell Bio, Shanghai, China), 1% penicillin-streptomycin (Cat. #15140-122, Thermo Fisher Scientific, Waltham, MA, USA), and maintained in a humidified atmosphere with 5% CO_2_ at 37 ℃. The osimertinib-resistant (Osi-R) cells were generated by gradually adding osimertinib (Cat. #S7297, Selleck, Houston, TX, USA) to PC9 and HCC827 cells at concentrations ranging from 1 nmol/L to 1 μmol/L. After three to four months of induction, the IC_50_ value of osimertinib exceeded 1 μmol/L, indicating the successful induction of Osi-R cell lines.

### Western blotting assay

The treated cells were lysed using RIPA buffer (Cat. #P0013B, Beyotime, Shanghai, China) containing protease inhibitor cocktail (Cat. #4693116001, Roche, Switzerland). The proteins were separated by 10% SDS-PAGE and transferred to 0.45-μm membranes (Cat. #IPVH00010, Millipore, Merck, Germany). The membranes were incubated overnight with primary antibodies against E-cadherin (1∶1000, Cat. #3195, Cell Signaling Technology [CST], Danvers, MA, USA), N-cadherin (1∶1000, Cat. #22018-1-AP, Proteintech, Wuhan, Hubei, China), vimentin (1∶1000, Cat. #5741, CST), vascular endothelial growth factor A (VEGFA; 1∶1000, Cat. #19003-1-AP, Proteintech), p-Akt (1∶1000, Cat. #4060, CST), and Akt (1∶1000, Cat. #9272, CST), and β-actin (1∶5000, Cat. #66009-1-Ig, Proteintech). The membranes were then incubated with the appropriate secondary antibodies (Cat. #7074 and #7076, CST), followed by exposure to a horseradish peroxidase-based luminescent solution (Cat. #WBKLS0500, Millipore).

### Enzyme-linked immunosorbent assay (ELISA)

A human VEGFA ELISA kit (Cat. #FMS-ELH-046, FCMACS, Nanjing, China) was used to determine the VEGFA levels in the cell culture supernatants of HCC827-Osi-R (HCC827-OR) cells. The supernatant was collected, and the cell fragments were removed by centrifugation at 1500 rpm for 10 min. It was stored at −80 ℃ for subsequent experiments, following the manufacturer's instructions.

### RNA extraction and real-time reverse transcription-PCR (qRT-PCR)

The total RNA from treated cells was extracted using TRIzol reagents (Cat. #R401-01, Vazyme, Nanjing, China). The RNA was then reverse-transcribed into cDNA using a transcription kit (Cat. #R323-01, Vazyme) following the manufacturer's protocol. Subsequently, the qRT-PCR assay was performed using SYBR Green Master Mix (Cat. #Q411-02, Vazyme). The relative expression levels of the target genes were analyzed using the 2^−∆∆Ct^ method. The primer sequences for qRT-PCR were as follows: *VEGFA* forward: 5*'*-CCCACTGAGGAGTCCAACAT-3*'*, *VEGFA* reverse: 5*'*-TCCCTTTCCTCGAACTGATT-3*'*, *GAPDH* forward: 5*'*-GAAGGTGAAGGTCGGAGTC-3*'*, and *GAPDH* reverse: 5*'*-GAAGATGGTGATGGGATTTC-3*'*.

### Methylthiazolyldiphenyl-tetrazolium bromide (MTT) assay

PC9, HCC827, PC9-Osi-R (PC9-OR), and HCC827-OR cells were seeded into 96-well plates at a density of 3 000–5 000 cells/well. The next day, the cells were cultured with varying concentrations of osimertinib and anlotinib (a generous gift from Chia Tai Tianqing Pharmaceutical Group Co., Ltd., Nanjing, China). After 48 h of incubation, the cells were incubated with a culture medium containing MTT for 2–4 h. Subsequently, formazan was dissolved using dimethyl sulfoxide (DMSO). Finally, the absorbance was measured at 490 nm, and the IC_50_ value was calculated.

### Colony formation assay

PC9, HCC827, PC9-OR, and HCC827-OR cells in the log phase were seeded into six-well plates at a density of 1 000 cells/well. The next day, the cells were cultured with a medium containing osimertinib and anlotinib if required for 10–14 days. Upon the formation of colonies containing more than 50 cells, the cells were fixed with methanol for 15 min, stained with Giemsa (Cat. #C0131, Beyotime) for 45 min, and visualized under a microscope.

### Wound-healing assay

The treated PC9, HCC827, PC9-OR, and HCC827-OR cells were seeded in 6-well plates. A 100-μL pipette tip was used to scratch the cell monolayer when the cells grew to around 80% confluence. In the wound-healing assay, the scratches were recorded at 0 h and 24 h by using a microscope, and the wound healing conditions were analyzed after 24 h of culture.

### Transwell assay

The PC9, HCC827, PC9-OR, and HCC827-OR cells in the logarithmic growth phase were seeded in the Transwell chamber (Cat. #3422, Corning, New York, USA) at a density of 5 × 10^4^ cells per well. Serum-free RPMI 1640 medium was added to the upper chamber, while the lower chamber was supplemented with 1640 medium containing 20% FBS for 48 h. The number of cells penetrating the membrane was observed by using a microscope and photographed. When needed, 1 μmol/L osimertinib and 4 μmol/L anlotinib were added.

### Tube formation assay

A total of 50 μL precooled Matrigel (Cat. #356234, Corning, USA) was added to 96-well plates and incubated at 37 ℃ for 30 min. The supernatant of the indicated cells was then extracted, and a mixture of the supernatant and human umbilical vein endothelial cells (HUVECs) at a density of 1.5 × 10^4^ cells was added to each well. The cells were then incubated at 37 ℃ for 6 h. Subsequently, the tube-like structures were observed under a microscope.

### Immunofluorescence staining

The treated cells (PC9, PC9-OR, HCC827, and HCC827-OR) were fixed with 4% paraformaldehyde and permeabilized with Triton X-100. Then, the cells were blocked and incubated overnight at 4 ℃ with the indicated primary antibodies. On the next day, the cells were incubated with fluorescein (FITC)-conjugated Affinipure Goat anti-Rabbit IgG antibody (1∶200, Cat. #SA00003-2, Proteintech) at 37 ℃ for 1 h and counterstained with DAPI (Cat. #C1006, Beyotime) for 5 min. Subsequently, the fluorescence signals were detected by using a fluorescence microscope (Zeiss, Oberkochen, Germany).

For phalloidin staining, cells were fixed with 4% paraformaldehyde, permeabilized with Triton X-100, incubated with phalloidin (Yeasen, Cat. #40786ES75, Shanghai, China) for 30 min, and counterstained with DAPI for 10 min. Then, the fluorescence signals were detected using the Nikon A1R confocal microscope (Nikon, Japan).

### Patient information

Three NSCLC patients exhibiting resistance to osimertinib were selected from the Affiliated Jinling Hospital, Nanjing Medical University. The experiments involving these patients were approved by the Institutional Review Board of the Affiliated Jinling Hospital, Nanjing Medical University (No. 2023DZGZR-030).

### Xenograft experiment

A total of 20 male nude mice (BALB/c) aged 4–6 weeks were procured from GemPharmatech (Nanjing, China) for xenograft experiments. The well-grown PC9-OR cells (3 × 10^6^ cells) were resuspended in 50 μL PBS, mixed with 50 μL of Matrigel, and inoculated on the inner side of the left upper limb of the nude mice. When the transplanted tumors reached the dimensions of more than 50 mm^3^ on the fifth day, these nude mice were randomly divided into four groups based on the tumor size, with five mice in each group. Each group underwent daily intragastric administration of either PBS, osimertinib (5 mg/kg), anlotinib (3 mg/kg), or a combination of osimertinib and anlotinib for 14 days. The mice were weighed and measured for tumor sizes every three days. Tumor volume was calculated using the formula: L (length) × W (width)^2^/2. The nude mice were euthanized after 14 days of administration. The tumors were excised, weighed, fixed with paraformaldehyde, and embedded with paraffin for subsequent IHC experiments. Animal experiments were approved by the Animal Ethics Committee of the Affiliated Jinling Hospital, Nanjing Medical University (No. 2023JLHGZRDWLS-00032).

### Immunohistochemistry (IHC) assay

Paraffin sections were used for IHC experiments on samples from three patients and twenty mice. The slides were antigen-retrieved and incubated overnight with E-cadherin (1∶200, Cat. #3195, CST), N-cadherin (1∶1000, Cat. #66219-1-Ig, Proteintech), vimentin (1∶200, Cat. #5741, CST), VEGFA (1∶500, Cat. #19003-1-AP, Proteintech), and Ki-67 (1∶300, Cat. #ab15580, Abcam, Cambridge, UK), respectively. Then, the slides were incubated with horseradish peroxidase-conjugated secondary antibodies, and the indicated proteins were visualized using diaminobenzidine (DAB, Cat. #K3468, Dako, Denmark). The sections were counterstained with hematoxylin and dehydrated with graded ethanol before being sealed for microscopic observations. The IHC staining density was scored as negative or weakly positive (score 1), moderately positive (score 2), or strongly positive (score 3). The percentage of positive cancer cells was classified as 1 (< 25%), 2 (25%–50%), 3 (50%–75%), and 4 (> 75%). An H-score was calculated by multiplying the intensity score and the proportion score to quantitatively evaluate the protein expression.

### TdT-mediated dUTP nick end labeling (TUNEL) assay

For the TUNEL experiment, the paraffin sections of mouse samples were deparaffinized and subsequently processed according to the manufacturer's instructions (Cat. #A112, Vazyme). After DAPI counterstaining, the apoptotic signals were observed using a microscope and photographed for subsequent statistical analysis.

### Statistical analysis

The intensity of the Western blotting bands was quantified using ImageJ software. The Student's *t*-test was used to analyze the quantitative data. Two-way ANOVA was used to compare differences among more than two groups. The data were expressed as the means ± standard deviation. Moreover, GraphPad Prism software was used to generate the graphs. A significant difference was indicated as ^*^P < 0.05, ^**^P < 0.01, and ^***^P < 0.001. All experiments were performed in triplicate.

## Results

### Anlotinib reversed clinical osimertinib resistance and EMT formation in Osi-R patients

**Figure 1:**
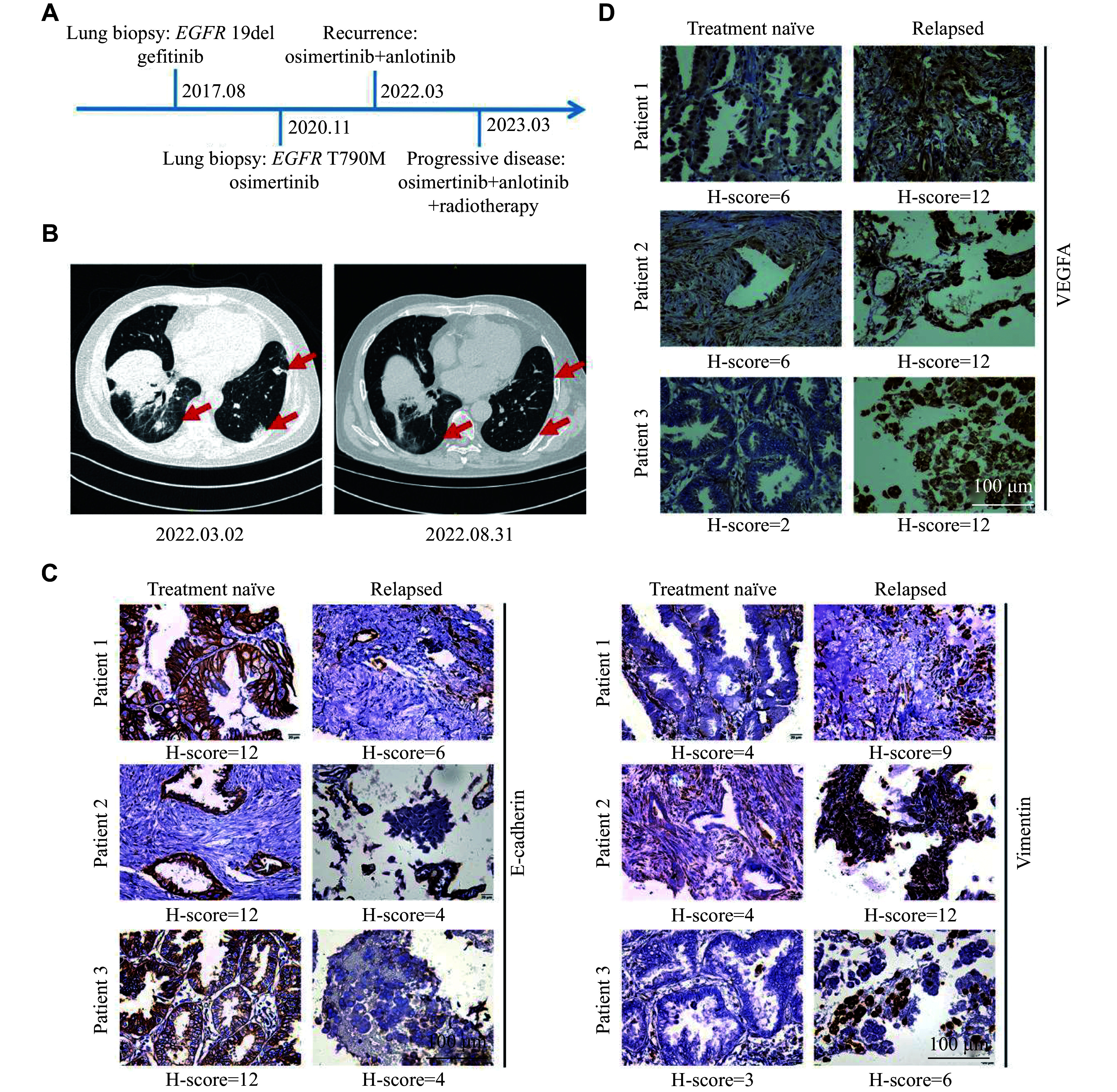
Combination therapy of osimertinib and anlotinib was effective for osimertinib-resistant (Osi-R) patients. A: A patient with *EGFR* T790M mutation developed osimertinib resistance and benefited from the combination therapy with anlotinib. B: The computed tomography imaging of the patient before osimertinib treatment and after developing osimertinib resistance. C and D: Representative images of the immunohistochemistry (IHC) staining of E-cadherin and vimentin (C) as well as VEGFA (D) in naïve and relapsed tissues of patients with acquired osimertinib resistance. H-scores are listed individually. Scale bar = 100 μm.

It is known that cancer patients may develop resistance to osimertinib over time. In a case study, a 57-year-old male was diagnosed with lung cancer at stage T4N0M1a and underwent a lung biopsy in August 2017. The patient exhibited an *EGFR*-19del mutation, as revealed by the next-generation sequencing, and received oral gefitinib for targeted therapy. The patient switched to osimertinib in November 2020 because of the emergence of the *EGFR* T790M mutation. However, the patient experienced recurrence in March 2022, and the treatment regimen was modified to a combination of osimertinib and anlotinib (***Fig. 1A***). The computed tomography imaging revealed a notable reduction in tumor size in the lungs in August 2022, compared with that in March 2022, particularly on the left side (***Fig. 1B***). This demonstrated the synergistic antitumor effects of osimertinib and anlotinib combination therapy in the case of osimertinib resistance.

Increasing studies have focused on the mechanisms of osimertinib resistance, but mechanisms and treatment options targeting EMT, one of the bypass mechanisms of osimertinib resistance, have received less attention from investigators, compared with other resistance mechanisms. To explore the specific metastatic mechanisms in lung cancer patients with osimertinib resistance, three patients who underwent a secondary lung biopsy at the Affiliated Jinling Hospital, Nanjing Medical University, were recruited. Paraffin sections of lung tissues were used for IHC experiments. The results indicated a decrease in the expression levels of E-cadherin but an increase in the expression levels of vimentin in the lung tissues of these three patients upon developing resistance (***Fig. 1C***). Furthermore, neovascularization is a crucial process during oncogenesis, in which VEGFA enhances the formation of blood vessels. In the present study, the expression of VEGFA was also upregulated in the recurrent tumor slides (***Fig. 1D***). All the H-scores are provided in ***Fig. 1C*** and ***1D***.

### Establishment and identification of PC9-OR and HCC827-OR cells

**Figure 2:**
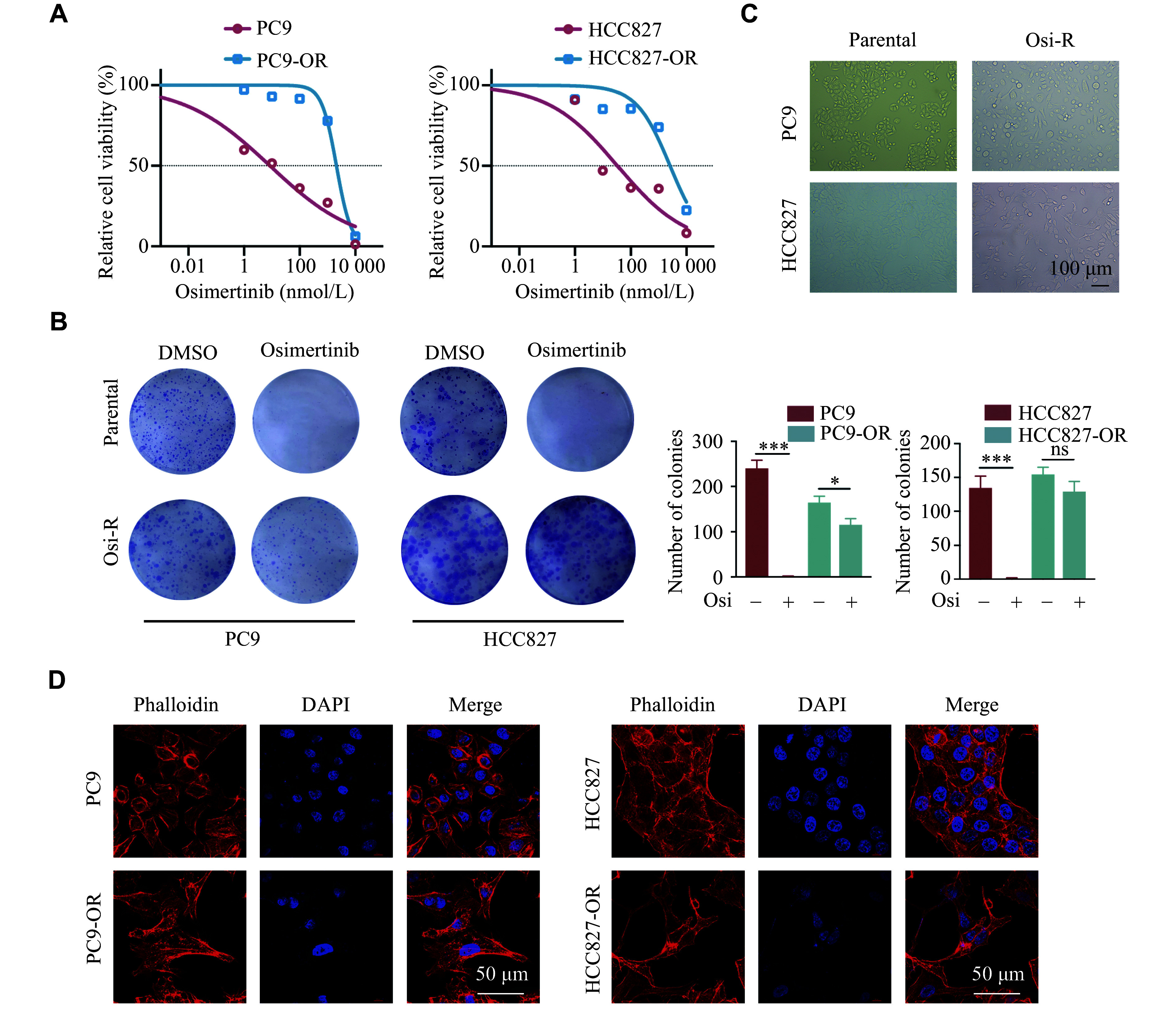
Establishment of PC9-OR and HCC827-OR cells from parental cells. A: PC9, PC9-OR, HCC827, and HCC827-OR cells were treated with osimertinib at the concentrations of 0, 1, 10, 100, 1000, and 10000 nmol/L for 48 h. The MTT assay was performed to determine the half-maximal inhibitory concentration (IC_50_) values of osimertinib. B: PC9 and HCC827 cells, as well as their respective Osi-R cells, were treated with 200 nmol/L osimertinib for 10–14 days. Colony formation experiments were performed to evaluate the osimertinib resistance. The number of clones was statistically analyzed by Student's *t*-test. ^*^P < 0.05 and ^***^P < 0.001. C: Morphological changes in the parental and Osi-R cells of PC9 and HCC827. Scale bar = 100 μm. D: Parental and Osi-R cells were stained with phalloidin. Red indicates phalloidin staining; blue indicates the nucleus. Scale bar = 50 μm. The experiments were performed in triplicate. Abbreviations: ns, no significance; OR and Osi-R, osimertinib-resistant.

To elucidate the mechanisms and clinical treatment strategies associated with the EMT-induced osimertinib resistance, we performed a series of experiments by stimulating PC9 and HCC827 cells with osimertinib starting at a concentration of 1 nmol/L. Upon observing cell viability even at 1 μmol/L osimertinib, we further treated the PC9, PC9-OR, HCC827, and HCC827-OR cells with osimertinib at various concentrations. The 48-h MTT assay results showed that the IC_50_ values of osimertinib in PC9 and PC9-OR cells were 8.54 nmol/L and 2.10 μmol/L, respectively. Similarly, the IC_50_ values of osimertinib were 33.59 nmol/L and 2.58 μmol/L in the HCC827 and HCC827-OR cells, respectively (***Fig. 2A***), indicating the successful induction of Osi-R cell lines. Furthermore, colony formation experiments demonstrated that PC9 and HCC827 parental cells failed to form colonies when treated with 200 nmol/L osimertinib, while the colony formation ability of PC9-OR and HCC827-OR cells remained unaffected by the same concentration of osimertinib (***Fig. 2B***). This observation suggested that PC9-OR and HCC827-OR cells exhibited resistance to osimertinib. Notably, a gradual acquisition of EMT phenotypes was observed in the PC9-OR and HCC827-OR cells during the induction of Osi-R cells, indicating an increased tendency towards mesenchymalization (***Fig. 2C***). Additionally, morphological changes were identified using phalloidin staining and confocal microscopy. Accordingly, the results showed that the filopodia of PC9-OR and HCC827-OR cells were longer than those of the parental cells, suggesting a higher invasive ability of these Osi-R cells (***Fig. 2D***).

### EMT of PC9-OR and HCC827-OR cells

**Figure 3:**
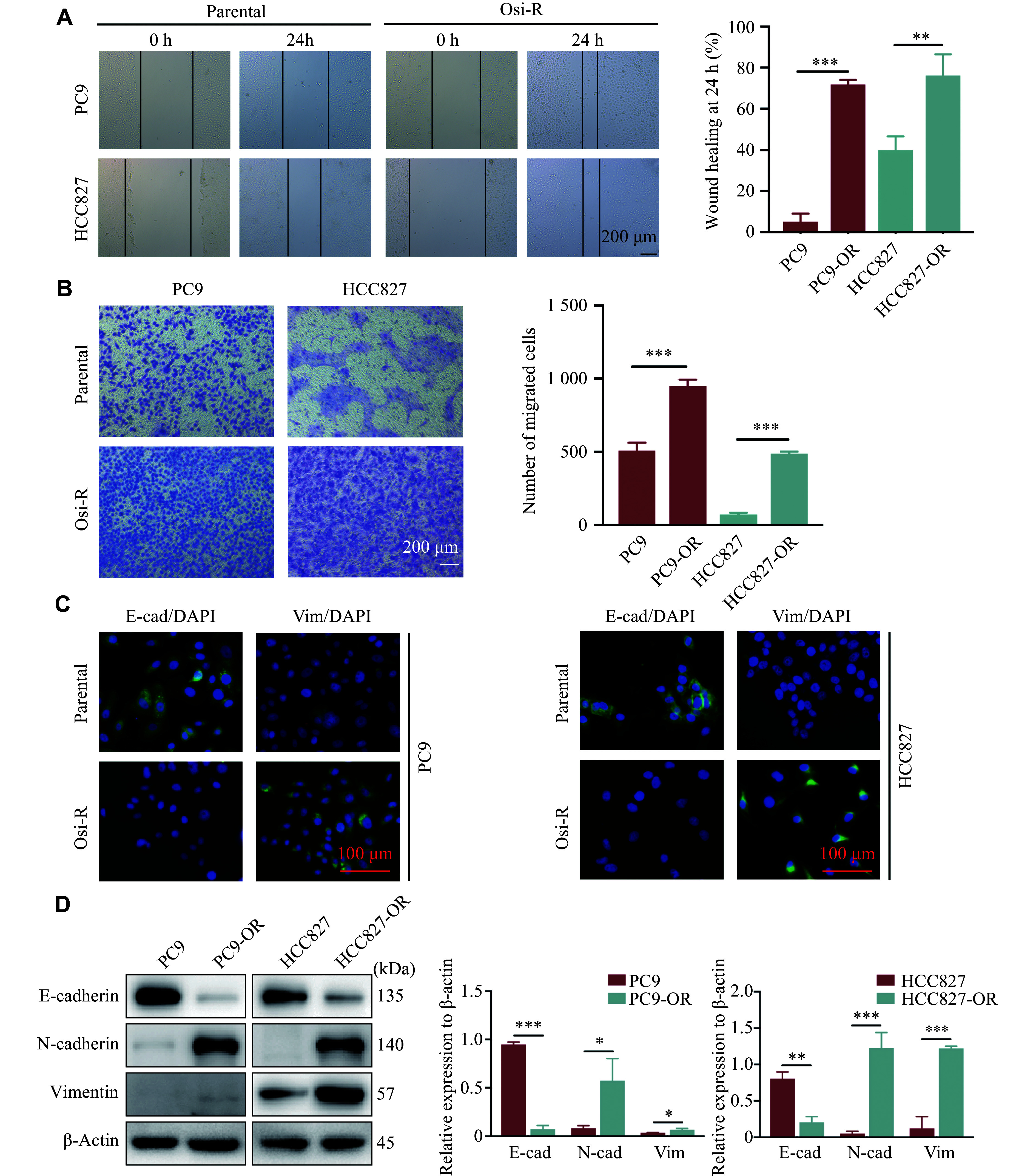
PC9-OR and HCC827-OR cells exhibited EMT properties. A and B: Wound-healing (A) and Transwell assays (B) were performed to determine the migratory abilities of Osi-R cells. Scale bar = 200 μm. C and D: Immunofluorescence staining (C) and Western blotting (D) experiments showed the expression of EMT markers in the parental and Osi-R cells. Scale bar = 100 μm. The experiments were performed in triplicate. The Student's *t*-test was used to analyze the quantitative data. ^*^P < 0.05, ^**^P < 0.01, and ^***^P < 0.001. Abbreviations: OR and Osi-R, osimertinib-resistant; E-cad, E-cadherin; Vim, vimentin.

The EGFR-independent pathways include bypass activation, small-cell transformation, and EMT^[8]^. To verify EMT in the Osi-R cells, we performed wound-healing and Transwell assays, and found an enhanced migratory ability of the PC9-OR and HCC827-OR cells, compared with the parental cells (***Fig. 3A*** and ***3B***). Furthermore, the immunofluorescence experiment indicated a stronger fluorescence signal of E-cadherin but a weaker fluorescence signal of vimentin in the parental cells than in the Osi-R cells (***Fig. 3C***). Moreover, Western blotting experiments showed decreased expression levels of E-cadherin but increased expression levels of N-cadherin and vimentin in the PC9-OR and HCC827-OR cells compared with their parental cells (***Fig. 3D***). These findings indicated that the PC9-OR and HCC827-OR cells might enhance their migratory ability through EMT, ultimately leading to osimertinib resistance.

### Anlotinib exhibited a synergistic effect in overcoming osimertinib resistance in PC9-OR and HCC827-OR cells

**Figure 4:**
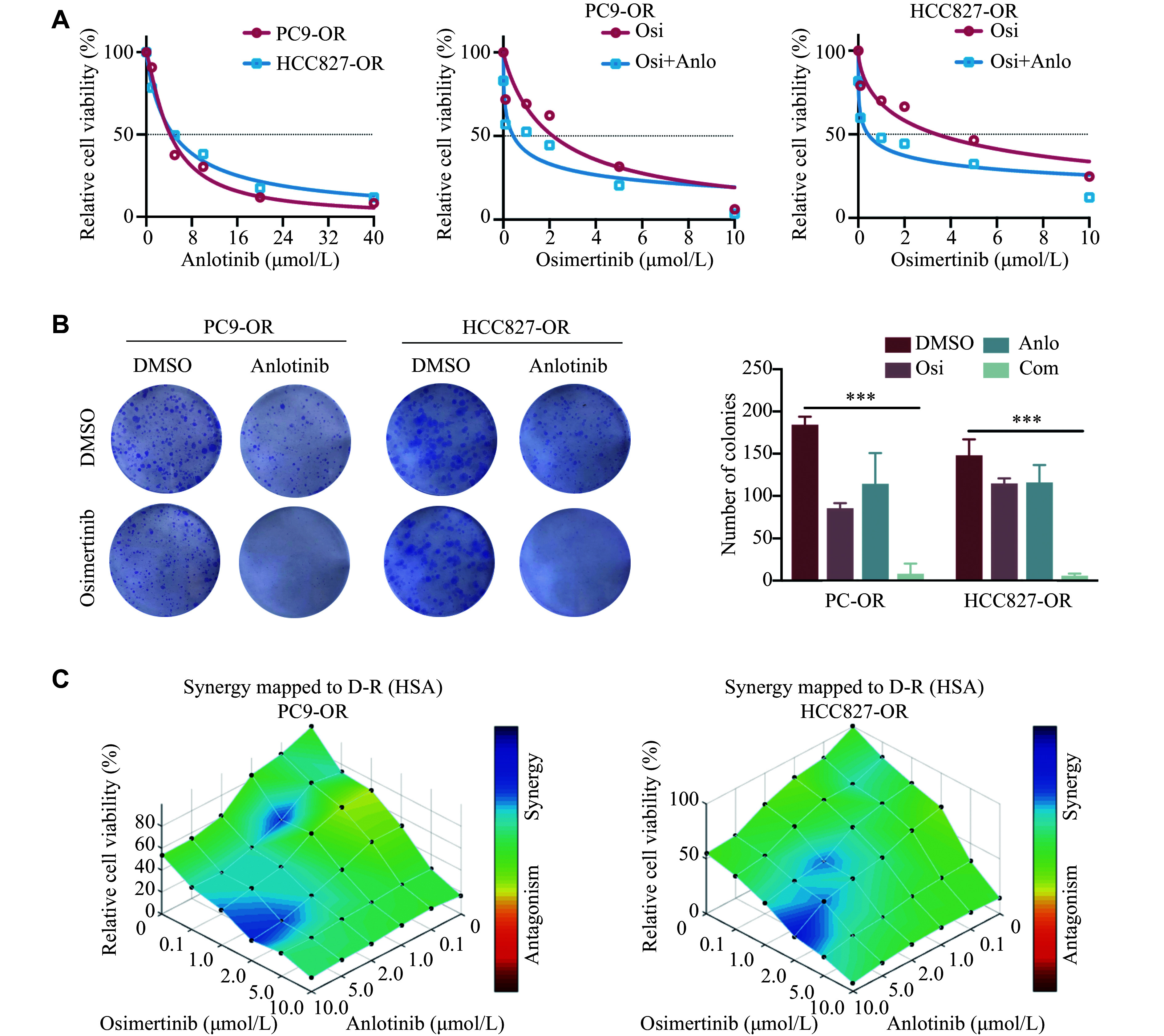
Combination therapy with anlotinib reversed osimertinib resistance. A: Osimertinib-resistant cells were treated with anlotinib (0, 1, 5, 10, 20, and 40 μmol/L), or osimertinib (0, 0.1, 1, 2, 5, and 10 μmol/L) and anlotinib (1 μmol/L) for 48 h. The MTT assays were performed to determine the IC_50_ values of osimertinib and anlotinib. B: PC9-OR and HCC827-OR cells were cultured with osimertinib at 200 nmol/L in the presence or absence of 1 μmol/L anlotinib for 10–14 days. Colony formation experiments were performed to verify the combination effect. C: PC9-OR and HCC827-OR cells were treated with osimertinib and anlotinib for 48 h. MTT assays were performed to determine cell viability. Combenefit was used to verify the synergistic effect of osimertinib and anlotinib. The experiments were performed in triplicate. Two-way ANOVA was used to compare differences among more than two groups. ^***^P < 0.001. Abbreviations: OR, osimertinib-resistant; Osi, osimertinib; Anlo, anlotinib; Com, combination of osimertinib with anlotinib.

To further explore whether anlotinib reverses osimertinib resistance by inhibiting EMT, we performed MTT assays in PC9-OR and HCC827-OR cells using various concentrations of osimertinib with or without anlotinib. The results showed that the IC_50_ value of anlotinib was 4.199 μmol/L in PC9-OR cells and 4.775 μmol/L in HCC827-OR cells. Moreover, the IC_50_ value of osimertinib in PC9-OR cells significantly decreased from 2.15 μmol/L to 0.43 μmol/L after the addition of anlotinib. Similarly, the IC_50_ value of osimertinib in HCC827-OR cells decreased from 3.41 μmol/L to 0.43 μmol/L with anlotinib treatment (***Fig. 4A***). Moreover, colony formation experiments revealed that osimertinib treatment alone failed to inhibit the colony formation of PC9-OR and HCC827-OR cells; however, when combined with anlotinib, the colony formation of Osi-R cells was significantly inhibited (***Fig. 4B***). The results of MTT and colony formation experiments collectively supported the conclusion that anlotinib could effectively reverse osimertinib resistance in PC9-OR and HCC827-OR cells. To further validate the synergistic effect of osimertinib and anlotinib, we performed an MTT assay with six individual concentrations of osimertinib and anlotinib, and analyzed the data using Combenefit, an interactive platform for analyzing and visualizing drug combinations, which revealed a consistent synergistic effect of osimertinib and anlotinib at several concentrations (***Fig. 4C***). Therefore, 1 μmol/L osimertinib combined with 4 μmol/L anlotinib was used for the subsequent experiments.

### Anlotinib reversed EMT and tube formation in Osi-R cells

**Figure 5:**
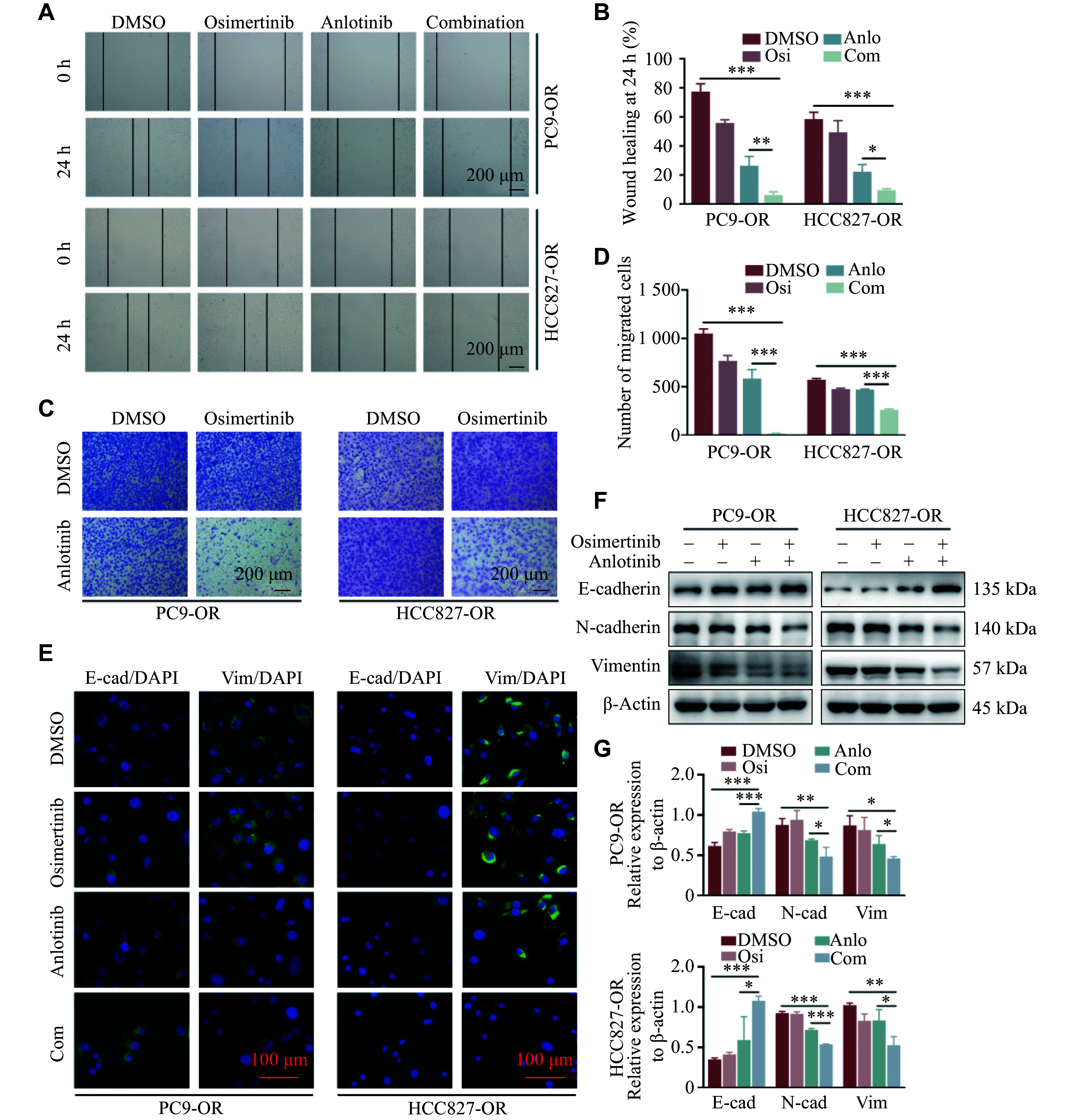
Combination of osimertinib and anlotinib reversed the EMT in resistant cells. A–G: PC9-OR and HCC827-OR cells were treated with osimertinib (1 μmol/L), anlotinib (4 μmol/L), or the combination for 24 h. Wound-healing (A and B) and Transwell (C and D) assays were performed to determine the migratory abilities. Scale bar = 200 μm. E–G: Osi-R cells were treated with osimertinib, anlotinib, or the combination. Immunofluorescence staining was performed to determine the expression levels of E-cadherin and vimentin (E). Scale bar = 100 μm. Western blotting analyses were performed to determine the expression levels of the EMT markers, *i.e.*, E-cadherin, N-cadherin, and vimentin (F and G). The experiments were performed in triplicate. The Student's *t*-test was used to statistically analyze the quantitative data. Two-way ANOVA was used to compare differences among more than two groups. ^*^P < 0.05, ^**^P < 0.01, and ^***^P < 0.001. Abbreviations: OR and Osi-R, osimertinib-resistant; E-cad, E-cadherin; Vim, vimentin; ns, no significance; Osi, osimertinib; Anlo, anlotinib; Com, combination of osimertinib with anlotinib.

To investigate whether the combination of osimertinib and anlotinib could inhibit the migratory ability of Osi-R cells, we performed wound-healing and Transwell assays. The results revealed that this combination effectively inhibited the migratory ability of PC9-OR and HCC827-OR cells, ultimately enhancing their sensitivity to osimertinib (***Fig. 5A***–***5D***). Furthermore, the immunofluorescence assays revealed a significant upregulation of E-cadherin and downregulation of vimentin with combination treatment (***Fig. 5E***). To validate these results, we treated the two Osi-R cell lines with 1 μmol/L osimertinib, with or without 4 μmol/L anlotinib, for 24 h. Western blotting analysis indicated that the combined strategy significantly inhibited the expression levels of N-cadherin and vimentin while upregulating E-cadherin expression. This finding strongly suggested that adding anlotinib to osimertinib inhibited EMT formation in Osi-R cells (***Fig. 5F*** and ***5G***). Thus, the underlying mechanism of anlotinib in reversing osimertinib resistance in PC9-OR and HCC827-OR cells involved the inhibition of EMT formation.

**Figure 6:**
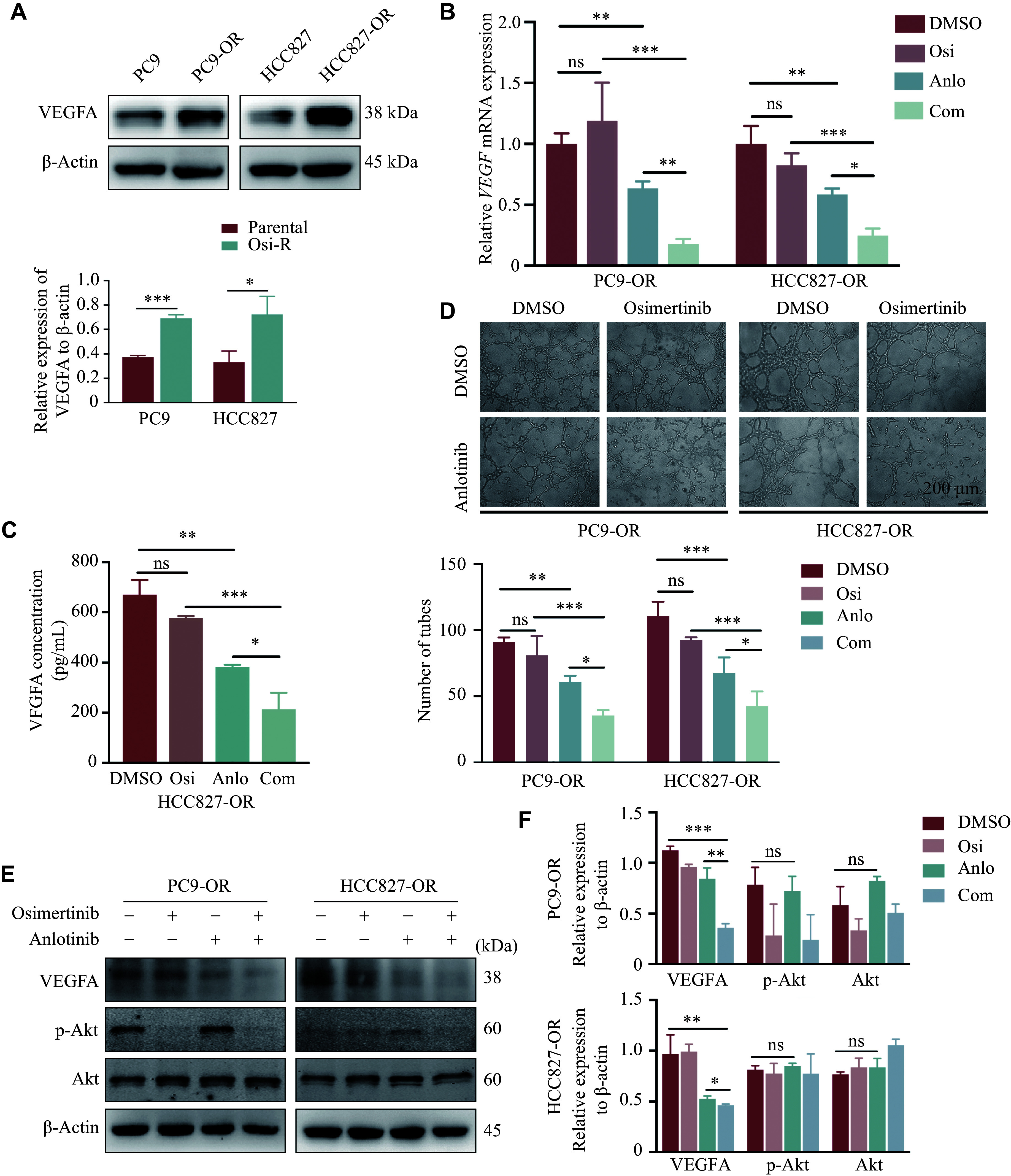
VEGFA was highly expressed in Osi-R cells, and combined therapy inhibited VEGFA expression and tube formation. A: Expression levels of VEGFA in both Osi-R cells and their parental cells were determined by Western blotting. B–F: PC9-OR and HCC827-OR cells were treated with osimertinib (1 μmol/L), anlotinib (4 μmol/L), or the combination for 24 h. mRNA levels of VEGFA in Osi-R cells were determined by qRT-PCR (B). Expression levels of VEGFA in HCC827-OR cells were determined by ELISA (C). Tube formation experiments showed tube-like structures in Osi-R cells treated with osimertinib, anlotinib, or the combination. Scale bar = 200 μm (D). Protein levels of VEGFA and p-Akt in the Osi-R cells treated with osimertinib, anlotinib, or the combination were determined by Western blotting (E and F). The experiments were performed in triplicate. The Student's *t*-test was used to statistically analyze the quantitative data. Two-way ANOVA was used to compare differences among more than two groups. ^*^P < 0.05, ^**^P < 0.01, and ^***^P < 0.001. Abbreviations: OR and Osi-R, osimertinib-resistant; E-cad, E-cadherin; Vim, vimentin; ns, no significance; Osi, osimertinib; Anlo, anlotinib; Com, combination of osimertinib with anlotinib.

The mechanism of anlotinib in NSCLC involves anti-angiogenesis^[15]^. To further investigate whether anlotinib could reverse resistance *via* anti-angiogenesis in the Osi-R cells, we examined the expression levels of VEGFA in these Osi-R cells. The results showed that VEGFA levels in Osi-R cells were significantly higher than those in their parental cells (***Fig. 6A***). The Osi-R cells were then treated with 1 μmol/L osimertinib and 4 μmol/L anlotinib for 24 h. Subsequently, the supernatants and cells were collected for ELISA and qRT-PCR assays, respectively. The results showed that the expression of VEGFA was significantly suppressed in both Osi-R cells by the combined treatments, compared with osimertinib or anlotinib treatment alone (***Fig. 6B*** and ***6C***). Moreover, tube formation experiments also showed that tube-like structures were scarcer in the combination group than in the other groups (***Fig. 6D***). Additionally, Western blotting analysis showed that the protein levels of VEGFA were lower in the combination group than in the other groups (***Fig. 6E*** and ***6F***). The results also showed that anlotinib did not reduce the phosphorylation level of p-Akt, which further validated that the mechanistic effects of osimertinib combined with anlotinib might result from the inhibition of EMT and VEGFA pathways. These results suggested that the combination therapy inhibited both EMT and angiogenesis, ultimately contributing to the reversal of osimertinib resistance.

### Reversal of osimertinib resistance by the combination of osimertinib and anlotinib *in vivo*

**Figure 7:**
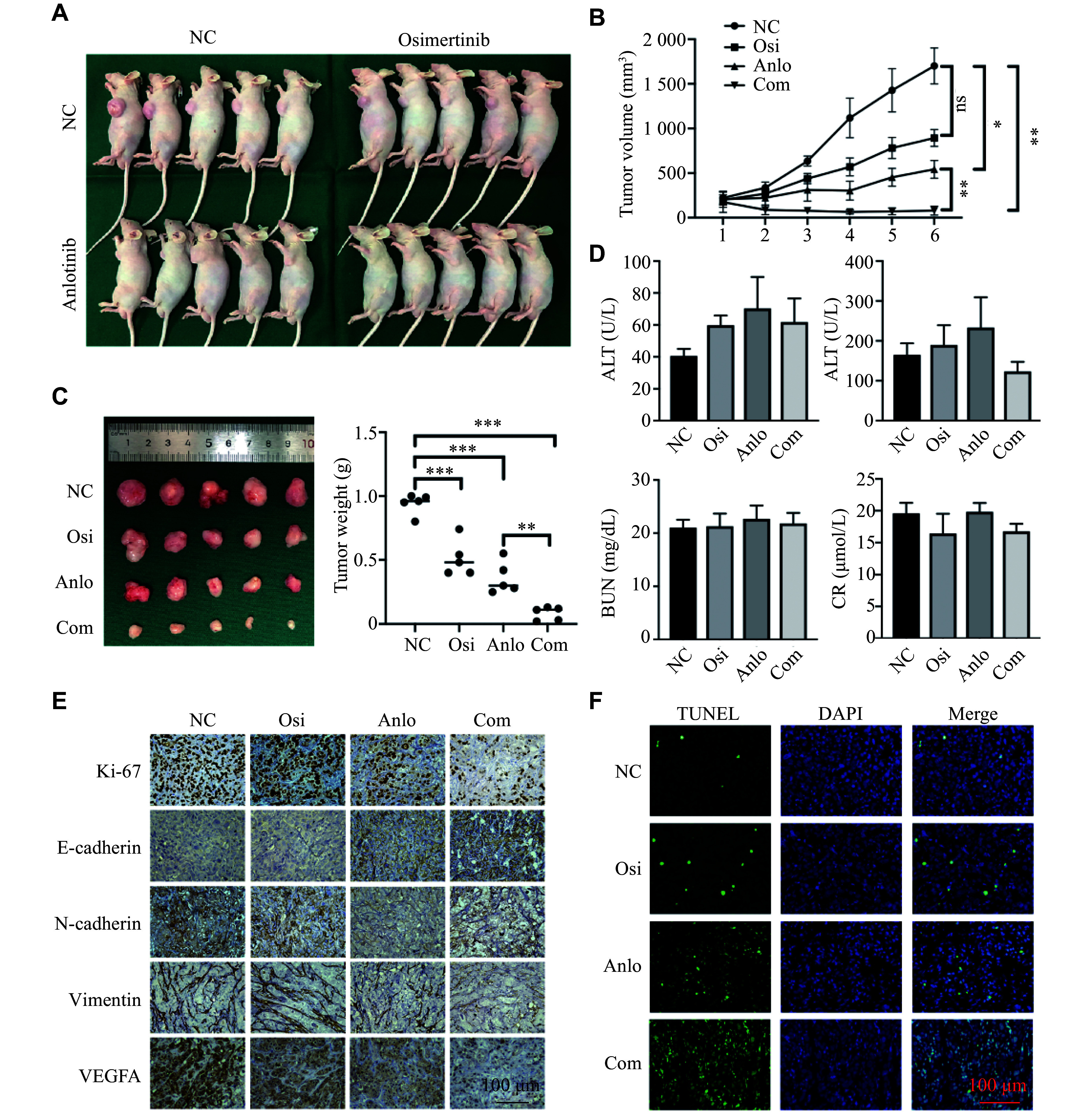
Anlotinib reversed osimertinib resistance *in vivo*. Nude mice were divided into four groups, with five mice in each group, and treated with 5 mg/kg osimertinib or 3 mg/kg anlotinib alone or in combination for 14 days. A: Photographs of mice in the four groups. B: The growth curves of tumors for mice in each group. C: Tumor volume and weight for mice in each group. D: The levels of aspartate transaminase (AST), alanine transaminase (ALT), blood urea nitrogen (BUN), and creatinine (Cr) in the serum were measured to determine the hepatic and renal function of mice. E: Representative images of IHC staining of Ki-67, E-cadherin, N-cadherin, vimentin, and VEGFA in the syngeneic tumors. Scale bar = 100 μm. F: The TUNEL assay for cell apoptosis was used to evaluate the synergistic effect of the combination therapy. Representative images are shown. Scale bar = 100 μm. The Student's *t*-test was used for statistical analysis. ^*^P < 0.05, ^**^P < 0.01, and ^***^P < 0.001. Abbreviations: NC, negative control; OR, osimertinib resistance; Osi, osimertinib; Anlo, anlotinib; Com, combination of osimertinib with anlotinib.

We further investigated whether osimertinib in combination with anlotinib could synergistically prevent NSCLC progression using xenograft experiments in mice (***Fig. 7A***). The growth curves showed greater inhibition of Osi-R xenograft growth in the mice treated with the combination of osimertinib and anlotinib, compared with osimertinib or anlotinib monotherapy (***Fig. 7B*** and ***7C***). Biochemical indicators were assessed and demonstrated the safety of the combinational strategy for mice, as no obvious impairments in hepatic and renal functions were observed in the indicated treatment groups (***Fig. 7D***).

Moreover, IHC experiments with the tumor tissues revealed that the positive signal of E-cadherin was the strongest in the combination group, while those of N-cadherin and vimentin were the weakest among the four groups, suggesting that combination therapy effectively inhibited EMT formation in tumor tissues *in vivo*, consequently reversing drug resistance. Simultaneously, this combined approach inhibited the expression levels of VEGFA, thereby suppressing the angiogenesis of tumors (***Fig. 7E***). Moreover, TUNEL experiments showed that the combined group exhibited the highest proportion of tumor apoptosis among the four groups, providing further evidence that the combination therapy exerted anti-tumor effects (***Fig. 7F***).

## Discussion

Targeting the EGFR pathway in anti-tumor therapies has demonstrated significant improvements in the survival rates of NSCLC patients^[16]^. Upon EGF binding to the extracellular domains of EGFR, receptor dimerization occurs, which mediates autophosphorylation, thereby activating multiple downstream signaling pathways, including PI3K/AKT and MAPK/ERK^[17–18]^. Abnormal EGFR activation causes sustained activation of genes associated with tumor proliferation and differentiation, thereby initiating and promoting tumor formation and progression^[19]^. To date, clinical therapies targeting EGFR encompass three generations of drugs. After an initial course of treatment with gefitinib or erlotinib, approximately half of the patients develop the T790M mutations^[20]^. The sequential administration of osimertinib can effectively inhibit this mutation^[21]^. With the advancement of clinical research, osimertinib has now been promoted to the frontline, significantly prolonging both the mOS and mPFS in patients^[22]^. In the FLAURA study, a randomized and double-blind phase Ⅲ clinical study that aimed to assess the efficacy and safety of osimertinib in previously untreated advanced NSCLC with EGFR mutations, it was found that, compared with first-generation EGFR-TKIs, first-line treatment with osimertinib significantly prolonged mPFS by 8.7 months, and extended mOS to 38.6 months, thereby reducing the mortality risk for patients by 20%^[23]^. The results from the ADAURA clinical study further emphasized the clinical use of osimertinib in resected EGFR-mutated NSCLC patients, revealing a 2-year PFS rate of 89% in the osimertinib group, compared with only 52% in the placebo group^[24]^. These findings underscore the significance of osimertinib in the management of *EGFR*-mutated NSCLC.

Recently, a series of studies have focused on overcoming osimertinib resistance to provide an effective clinical treatment for Osi-R patients^[25]^. While the fourth generation of EGFR-TKIs is still in development and not yet available in clinical practice, combination therapies after osimertinib resistance have shown promising potential in mitigating resistance^[26]^. Anlotinib, by inhibiting targets such as c-Kit (proliferation), RET (proliferation), FGFR (proliferation/metastasis), and c-Met (metastasis), has been demonstrated to control tumor cell proliferation and metastasis^[11]^. The mechanisms of osimertinib resistance include the activation of pathways, such as c-Met and FGFR, as well as the formation of EMT^[27]^. Moreover, anlotinib also exhibits inhibitory effects on these pathways^[28]^. A retrospective and exploratory study revealed a survival benefit of anlotinib in T790M-positive NSCLC patients with acquired osimertinib resistance^[29]^. Lei *et al*^[14]^ proposed that anlotinib could reverse osimertinib resistance by inhibiting the c-MET/MYC/AXL pathway. Furthermore, another study focusing on third-generation EGFR-TKIs indicated that anlotinib could reverse acquired resistance to gefitinib^[30]^. However, few investigations have explored whether anlotinib could reverse osimertinib resistance specifically by inhibiting the EMT pathway. The present study addressed this gap by revealing that tumor tissues of Osi-R patients tended to exhibit enhanced migration ability and elevated mesenchymal indicators. Therefore, we established resistant cell lines (PC9-OR and HCC827-OR) to demonstrate their enhanced metastatic ability using wound-healing and Transwell assays. Western blotting analysis revealed that the expression levels of mesenchymal phenotype indicators, such as N-cadherin and vimentin, were increased in the Osi-R cells, while the level of E-cadherin was reduced, compared with the parental cells. Thus, subsequent experiments were performed based on these Osi-R cells to explore the potential of anlotinib in inhibiting and reversing osimertinib resistance.

EMT is a process characterized by the loss of cellular polarity and adhesion, resulting in a shift towards a mesenchymal phenotype with enhanced migration capabilities^[31]^. Importantly, EMT serves as a resistance mechanism against various treatments, including targeted drugs, cytotoxic drugs, and radiotherapy^[32–33]^. The EMT process activates AXL through the PI3K/AKT pathway, leading to the loss of E-cadherin, thereby causing metastasis of NSCLC^[34]^. Furthermore, the increased expression of EMT transcription factors and ZEB1 expression may also lead to the EGFR-TKI resistance^[35]^. The co-inhibition of these pathways, along with the EGFR pathway, holds the potential to reverse the EMT-mediated resistance. Therefore, targeting EMT represents a promising strategy to effectively kill tumor cells and overcome resistance to EGFR-TKIs in NSCLC. In the present study, we observed that the combination of osimertinib and anlotinib effectively reversed osimertinib resistance induced by EMT *in vitro*. This finding was further validated in nude mice, where tumors in the combined treatment group exhibited the smallest size. The IHC results showed that E-cadherin was upregulated in the combination group, while the expression levels of N-cadherin and vimentin were reduced, indicating that the tumor cells underwent mesenchymal-to-epithelial transition. Based on these observations, we concluded that anlotinib effectively reversed osimertinib resistance, extending the duration of osimertinib treatment, and might consequently prolong the OS of patients by inhibiting the EMT occurrence. The current understanding of whether osimertinib resistance arises from the selection of pre-existing EMT clones or the secondary acquisition of a stromal phenotype by epithelial cells after EGFR-TKI treatment remains unclear. Future investigations targeting this mechanism and developing targeted treatment strategies may enhance treatment efficacy, offering clinical benefits for patients.

Briefly, the development of osimertinib resistance in NSCLC may be partly attributed to the occurrence of EMT. Anlotinib inhibits the migration of Osi-R cells, offering a potential solution to combat osimertinib resistance. The combination of osimertinib and anlotinib emerges as a promising clinical strategy for patients, holding the potential to extend their survival time.
